# Review of a nurse consultant’s role: Identifying the contribution made to people living with and beyond cancer

**DOI:** 10.1002/nop2.407

**Published:** 2019-11-26

**Authors:** Claire Taylor, Theresa Wiseman

**Affiliations:** ^1^ London North West University Healthcare NHS Trust Harrow UK; ^2^ Applied Health Research The Royal Marsden NHS Foundation Trust London UK

**Keywords:** cancer nursing, living with and beyond cancer, nurse consultant, practice development, Recovery Package, reflection, role evaluation

## Abstract

**Aim:**

To evaluate a new nurse consultant (NC) role, four specific objectives were set including examining the NC’s contribution to the local implementation over a 30‐month time period of the Recovery Package and assessing changes at a patient/professional/system level.

**Methods:**

An evaluative process was agreed using Donabedian's (2005) model for measuring the quality of care provided. It focused on the NC’s contribution to the Trust's LWBC agenda including a review of Recovery Package metrics, analysis of the NC template recording activity across different domains, 360‐degree feedback and personal reflections.

**Results:**

The evaluation demonstrated the NC’s influence on individual patient care with an increase in three of the four Recovery Package metrics to the Trust; improvements in delivery of services and a higher level of participation in activities aimed at raising awareness to LWBC at a regional and national level. Broader influences of this role were also evident.

**Impact**

- This paper provides a longitudinal account of the impact of the nurse consultant role and how it is enacted in a specific context to enhance care delivery to people living with and beyond cancer.
- It demonstrates that the evaluation of the nurse consultant role must not be viewed in isolation of the complex teams and systems that they work within to deliver patient care.

## 1 BACKGROUND

Nurse consultant (NC) roles aim to provide better outcomes for patients by improving services and quality (Department of Health, 1999). These posts were established to retain senior nurses midwifes and health visitors in practice, keep their clinical and managerial expertise at the bedside and provide leadership in their teams (Manley, 1997, 2002). They can help ensure the highest standards of care and effective treatment is provided (Stevenson Ryan & Masterson, 2011). They bridge expert nursing practice with learning, evaluation and measurement in the workplace, as well as providing clinical and political leadership (Giles, Parker, & Mitchell, 2014). The role demands extensive and highly specialized knowledge to deliver the following functions:

- An expert practice
- Professional leadership and consultancy
- Education, training and development
- Practice and service development
- Research and evaluation (Redwood, Carr, & Graham, 2005).

In 2000, the NHS modernization agenda stated that up to 5,000 consultant posts would be formed. Nearly two decades later, it seems the number of NC roles created is lower than envisaged, a recent cancer nurse workforce survey in England indicating less than 1% of nurses specializing in cancer are working in NC roles (47 WTE posts) (Macmillan Cancer Support, 2018). This suggests that decision‐makers underappreciate the value and worth of these roles (Franks, 2014). Yet England's Cancer Workforce Plan (Health Education England, 2017) highlights the need for a specialist cancer workforce in supporting the rising numbers of people now surviving cancer. Its ambition is to have enough skilled staff in the right areas to deliver the cancer strategy. It is therefore imperative that the contribution NCs can make to health care is articulated.

A key component of the cancer strategy is improving cancer survivorship – transforming support for people living with and beyond cancer (Cancer Research UK, 2015). Emerging evidence reveals the difficulties people can face when the treatment is over (Armes, Crowe, & Colbourne, 2009; Corner, Wagland, Glaser, & Richards, 2013; Glaser et al., 2013); one in four have to deal with the consequences of their treatment (Macmillan Cancer Support, 2013a) and up to a third consistently report problems associated with cancer and its treatment (Macmillan Cancer Support, 2013b). To address this, a package of interventions was designed to support people through their recovery including holistic needs assessment, a cancer care review, additional information on their treatment and after‐care to support self‐management and events to promote healthier lifestyles. Many organizations looked to nursing to help implement this “Recovery Package” (Henry, 2015).

At London North West Healthcare University NHS Trust, a Macmillan nurse consultant in Colorectal Cancer post was created with an expectation that the postholder would lead on cancer survivorship and implementation of the Recovery Package in addition to caring for those with complex needs. This was a new post so from the outset there was an expectation from the charity, Macmillan Cancer Support (who funded it for the first two years), that its impact would be evaluated.

## 2 METHOD

In line with recommendations by McSherry, Mudd, and Campbell (2007), the NC met with her manager and a Macmillan Development Manager before role commencement (March 2015) to decide on which data to collect with a view that these would subsequently be collated in a report to Macmillan. Their priority for review was the NC contribution to the Trust's “Living with and beyond cancer” (LWBC). A review period of 30 months from April 2015–September 2017 was chosen for pragmatic reasons. At the end of the data collection period, formal discussions with the Macmillan Partnership Quality Lead, Partnership Manager and Evaluation Lead took place to agree a framework for the evaluative process. The framework chosen was the Donabedian conceptual model for measuring quality care (2005). It has three components to it: structure, process and outcome. Structure effects process which in turn influences outcome, with each of these reflecting different attributes by which to evaluate – in this case, the service for those LWBC.

The evaluation questions were as follows:

1. How has the role contributed to Recovery Package implementation?
2. How has the role contributed to influencing at a regional, national and international level?
3. How did this NC post contribute towards outcomes at patient level/professional level/system level?
4. What difference does it make having a clinical person in a LWBC focussed role (in terms of “added value” of having a clinical background)?

The evaluation did not include other aspects of this NC’s role which must be acknowledged since a considerable proportion of the NC’s activity involved working to a complex colorectal cancer service giving direct patient care, managing 3 weekly team meetings/clinics, monitoring service delivery and making patient‐focused improvements to the cancer consequences’ service. These are outside the scope of this evaluation.

Four evaluation methods were used for this review:

### 2.1 Collection and analysis of Recovery Package activity

There are 4 main components to the Recovery Package which the Trust report quarterly activity on to the Cancer Vanguard (a partnership of 17 organizations working together to improve care across local cancer pathways). These include numbers of patients receiving the following:

- Holistic need assessments (HNA) – at two key points on the care pathway, within 30 days of diagnosis and 6 weeks of completing primary treatment.
- Treatment summary (TS) – prepared by the clinician delivering cancer treatment at the end of their treatment.
- A Health and Wellbeing event (HWBE) – offered to people who have completed treatment to learn about recovery, healthy lifestyle and supported self‐management.
- Numbers of breast, colorectal and urology patients entering the proposed new model of follow‐up care whereby those who are well can be stratified to a supported self‐management programme of after‐care.

These metrics were collated quarterly for the 18‐month period under examination and analysed for trends. These objective data helped answer questions 1 and 3.

### 2.2 Use of the NC template

The template developed by Gerrish et al (2011) was adopted for prospective data recording; its aim was to capture the personal, professional and organizational levels of influence the NC had. It enabled quarterly recording of clinical, educational and leadership activity over 30 months*.* The content of all 6 of the templates completed during this time was analysed – an example of one section of the detailed quarterly summaries can be seen in Table 1. These data assisted answer questions 2 and 3.

**Table 1:** Activity over time across the three main Recovery Package interventions for all primary cancer patients in the Trust

Recovery Package metrics	HNA X 2		Treatment summary, %	HWBE, %
Period	Within 31 days of diagnosis, %	Within 6 weeks, %		
Q1 2015/16	51	29	14	4
Q2 2015/16	44	33	10	2
Q3 2015/16	50	42	17	4
Q4 2015/16	38	30	11	1
Q1 2016/17	38	29	19	0
Q2 2016/17	58	38	7	0
Q3 2016/17	67	25	15	5
Q4 2016/17	42	18	28	5
Q1 2017/18	86	25	27	15

### 2.3 360‐degree feedback

The HS360 Nurse Leaders survey was selected to gain 360‐degree feedback. Thirteen individuals were purposively chosen who regularly interacted with the NC and would provide a breadth of representation from each of the following groups: managers, senior clinicians, peers (internal), colleagues (external) and patients. They were invited to anonymously complete the survey in January 2017 – by which stage the NC has been in post 20 months. The survey was comprised of the following six domains: Patient Focus and Quality; Clinical Expertise; Service Improvement and Change Management; Self‐Awareness and Impact on Others; and Leadership and Inspiring Others and Managing Services. A summary report was produced with highlights, lowlights and all respondents’ free‐text comments on the NC’s contribution. These external data provided insights which supported the answers to question 3 and to a lesser extent question 4.

### 2.4 Personal reflections

Using the template data, the impact of key LWBC service changes which the NC had either led or facilitated was charted over time to summarize LWBC service changes. A SWOT analysis was also completed to appreciate facilitating factors and intervening variables. These subjective data aimed to address all four questions. Research Ethics Committee approval was not required for this service evaluation.

## 3 RESULTS

The data collected through these four different evaluation methods are now presented to answer the four agreed evaluation questions in turn.

### 3.1 How has the NC role contributed to Recovery Package implementation?

The implementation of four different components of the Recovery Package (RP) to the Trust was objectively evaluated by analysing activity over 30 months. Data were collated each quarter, and 3 of the 4 components are presented in Table 1. Over the time period under review, HNA, TS and HWBE activity increased across all four of the metrics. The greatest increase in activity was seen in the completion of an HNA within 31 days diagnosis.

Benchmarking the Trust's RP activity was compared against the target set by the Cancer Vanguard that 70% of patients diagnosed with cancer will receive all the RP interventions. Table 1 reveals this target was only met for one of the 4 listed interventions – the completion of an HNA and care plan at diagnosis. However, the Trust's RP activity compares favourably with the other 11 Trusts in the Vanguard at Q1 2017/8, sitting in 5th position overall.

The fourth component of the RP was the number of patients entering on to a supported self‐management programme of after‐care. Over 90% of breast patients were entered on to this pathway which was above the target of 70% – a figure which was sustained over time. Whilst this was mainly due to the efforts of a proactive and experienced Lead Breast Care CNS, the NC supported this postholder providing advice and equipment. The colorectal and urology surveillance pathways were more complex to introduce than breast cancer after‐care, requiring the development of a safe IT monitoring system. IT project support and financial investment by the Trust became the main obstacles to the further development of these two pathways.

### 3.2 How did this NC role contribute to influencing at a more regional and national level?

Influencing at regional level occurred in three main ways; firstly, the NC was on the steering member of a regional LWBC pathway group which was a very dynamic partnership through this time, developing many useful resources, running educational events and disseminating information across the 14 Trusts to the Vanguard (at that time). The NC was able to use this membership to influence the Colorectal Cancer pathway group, acting as adviser to its members on LWBC issues and promoting cross‐fertilization of knowledge between the two pathway groups. This regional activity during the 30‐month period is summarized in Table 2.

**Table 2:** List of NC influencing at Cancer Vanguard regarding LWBC 2015–7

The NC influence in the regional LWBC Pathway group member included the following: Co‐organized a London‐based HWBE for 100 + people LWBC colorectal cancer Gave a clinical fora presentation on consequences of treatment Co‐led on the production of 3 documents: Guidance on Health and Wellbeing events – which was adopted Consequences of cancer position statement – circulated to Trusts in the Vanguard 3 HNA fact sheets – these are still available to all healthcare professionals to use across the LCA NC influence in the regional Colorectal Cancer pathway included the following Gave 2 clinical fora presentations: (a) Recovery Package and (b) stratified follow‐up Co‐produced the following documents: (a) treatment summary and (b) health and well‐being events for colorectal cancer patients Co‐facilitated 4 educational events for CRC nurses in the Vanguard to explain the Recovery Package and encourage its roll‐out Led on the production of guidelines for the management of chemotherapy‐induced peripheral neuropathy across Cancer Vanguard

A second influencing mechanism came through establishing a new regional service based at the Trust, for which the NC was the nurse lead. Her role in this is summarized in Table 3.

**Table 3:** List of activities regarding new regional service for people LWBC

**The NC influence in the development of the GI consequences’ service**
Organized the GI consequences’ service launch event for the service with help from Macmillan – regional leads in Gastroenterology and Oncology were invited Organized a GP educational event for the service for local GPs to attend Introduced patient information on common conditions diagnosed and had them approved by the Trust's communications’ department Co‐developed rectal bleeding score as a PROM Co‐developed sucralfate pathway to ensure patient care coordinated and any patient prescribed sucralfate is referred to the NC/senior nurse. Established patient database to monitor patients and record treatment outcomes

The third main way the NC was able to influence the LWBC agenda regionally was by sitting on the steering committee of the Rehabilitation group, Transforming Cancer Services in London (TCST), to influence priorities for cancer care across London. During this time, this group developed evidence and resources to directly improve cancer rehabilitation across London with a further aim to influence services commissioning.

At a national level, the NC was involved in raising nurses’ awareness to the needs of those LWBC and the associated support and interventions available through being a steering group member of the Cancer Nursing Partnership (CNP). The CNP was able to reach 20,000 UK nurses through producing monthly e‐bulletin journal articles and conference presentations. The NC was also Chair of the National Colorectal Cancer Nursing Network (NCCNN) through this time and invited key speakers on LWBC to both the 2016 and 2017 NCCNN annual conference.

Table 4 highlights the national activities the NC delivered during the review period.

**Table 4:** List of national activities in addition to membership of national groups

National presentations
In 2017, the NC gave 4 national conference presentations and one internal masterclass which featured LWBC
A joint abstract was accepted at ASCO, Survivorship Symposium in the USA
National publications
The NC co‐authored 5 papers with relevance to LWBC
National guidance
Invited section contributor to NICE’s national guidance (management of anterior resection syndrome – a consequence of CRC treatment)
The NC was invited to participate in one of the Cancer Task Force workshops, subsequently writing a joint letter to the Task Force to highlight the importance of addressing cancer survivors’ needs

### 3.3 How did this NC post contribute towards outcomes at patient level/professional level/system level?

The quarterly recording of the NC’s clinical, educational, leadership and research activity demonstrated the breadth and variety of ways the NC contributed to outcomes at patient, professional and system levels. See Table 5 for an illustration of activity across these four domains for one quarter at a patient level.

**Table 5:** NC metrics for patient activity Q4 2016/7

Level	Expert practice	Leadership	Teaching	Research
Patient	Continue to be key worker to complex colorectal cancer patients; 45 patients referred this quarter GI consequences’ service – 4 new patients and now working with OPD nurse manager to provide joint service	Recruited 1 more facilitators from team for the short HOPE Interviewed by national nursing journal regarding the GI nursing service – article in February issue	Organized and ran 2 HWBE Co‐facilitated a short HOPE programme – 13 patients 22.2.17	Collated Q3 LCA survivorship metrics

Additional insight of the NC activity is offered by specific examples at each of the three levels indicated by Gerrish, McDonnell, and Kennedy (2011):

1. At a patient level – the NC was spending 50%–70% time in clinical practice, much of the time supporting patients with significant consequences of being treated for cancer who therefore had complex needs. This might involve referring to other specialist services such as physiotherapy, welfare advice and pain clinics as well as working therapeutically with individuals to help them regain enough control and confidence in their recovery to self‐manage.
2. At a professional level – the NC provided consultancy to many CNSs on LWBC, for example mentoring a CNS working in Midlands who wanted to start a “Anterior Resection service” and also to national charities: Macmillan Cancer Support, Beating Bowel Cancer and Pelvic Radiation Disease Association (PRDA).
3. At a system level – the NC worked to her employing organization to ensure that LWBC became understood as an integral part of cancer care. This is evidenced through the changes presented in a chronological order in Table 6.

**Table 6:** Examples of service changes which had meaningful impact within the Trust 2015–2017

When	What happened	What impact did it have
June 2015	LWBC became a regular agenda item on monthly Cancer CNS team meeting	NC had opportunity to share knowledge, present metrics and offer support. LWBC become a familiar concept which all teams had to buy into
December 2015	HOPE programme facilitator training completed	NC and CNS started running the HOPE course, switching to the shorter version “HOPE: Taking control” in 2017 which runs 3 x year
January–October 2016	Development of Infoflex module in Trust	NC part of working group to develop IT solutions for RP implementation. By using this Infoflex module, CNSs could record their HNAs electronically rather than on paper
August 2016	Quarterly senior nurse LWBC meetings established	Created a forum for review and problem‐solving, developed a LWBC nursing strategy and enhanced peer support
September 2016	Recruited first full‐time LWBC Support Worker	Enabled delivery and evaluation of monthly HWBE and other patient events through the year
January–July 2017	Recruited 2 further LWBC Support Workers (SWs) to support CNSs	Supported development and delivery of HNA telephone clinics, streamlined reporting of HNA activity and maintenance of site‐specific databases for LWBC
August 2017	Established monthly SW LWBC meetings	Enabled discussion and collaboration which helped with the production of operational policies and a range of patient resources including a folder of local support agencies across the locality to support patient events

The 360‐degree feedback survey was conducted to gather objective feedback on the NC’s competencies and to then compare the NC’s self‐evaluation against each of the individual behavioural questions. All 13 people approached responded and completed the survey. The scoring system in this survey allows a score on each of the 48 items to a maximum of 5; scores of 4 represent very good and scores of 5 signal a clear strength, indicating an individual operates nearly always with a high level of effectiveness. Scores received were all rated as very good or above with a range of 4.43–4.79 and a median score of 4.67. External scores were higher than the self‐evaluation scores in 4 out of 6 of the domains.

The survey respondents perceived the NC to have the strongest competencies in having a patient focus (4.79) and providing clinical expertise (4.76) and lowest competencies in managing services (4.52). The scores for leadership were highest when acting as a role model (4.67) and inspiring others to work towards a shared vision (4.67) and lowest for having a visible presence (4.43). The free‐text comments (shown in Table 7) indicate the NC is perceived as fulfilling a leadership role.

**Table 7:** 360‐degree feedback – free‐text comments

Feedback source	Comment
Patient	Communicates thoughtfully, carefully and effectively
Patient	Very approachable. Makes time for you, regardless of how demanding her workload is. A true professional with a great balance of fairness and empathy
Senior	Clinical expert. Inspirational leader of service improvement. Researcher
Senior	Claire leads projects and gets results across organizational boundaries. She should be encouraged to develop this skill and do more of it
Peer	Communicates well and responds to patients’ needs and requirements
Peer	Claire has an amazing ability to complete a large volume of work in a timely manner. She encourages others to improve themselves
Junior	From my perspective, very direct, informative and gives advice and assistance as required. Has great passion for her specialist area whilst not losing sight of the patient
Junior	Claire Taylor is a visionary and a leader who works hard towards service improvement where patients receive evidence‐based and individualized care. She is fair and treats all staff and patients with respect and dignity. She is always there if the need arises

### 3.4 What difference does it make having a clinical person in a LWBC focussed role (in terms of “added value” of having a clinical background)?

The NC was a member of the CNS cancer team and spent over half the week delivering direct patient care. The team could observe her addressing LWBC issues with her patient group whilst also leading on LWBC events and initiatives. She therefore assumed both an individual and collective responsibility to deliver on the LWBC agenda.

The NC with support from the Lead Cancer Nurse was able to influence the clinical nurse specialist (CNS) contribution to the Recovery Package (RP) by making HNA a key performance indicator. She established and supported a forum to discuss the challenges in changing practice. It was through these conversations that CNSs in the team expressed frustrations about their difficulties performing the second HNA – planned to assess patient concerns after treatment, as many patients are transferred to other Trusts to receive cancer treatments. Fortunately, the NC was attuned to the complexity in patient pathways to the Trust and appreciated that for certain tumour sites it was not going to be possible to meet the 70% target of HNA completion for patients at this time.

In late August 2016, the NC detected anxiety amongst some team about delivering on the Recovery Package. The NC conducted a survey early in 2017 to understand this further and identified clinical overload as the most common perceived barrier to implementation. Some of the CNSs indicated they lacked time in their work plan to perform high‐quality HNA and then complete a detailed care plan. The NC was aware from her own practice that each HNA could take 30 min to complete and empathized with the clinical pressures each CNS service faced, but she noted the CNSs were (perhaps without realizing) regularly making detailed patient assessments which they could convert with relative ease into a formally recorded HNA. This led to open discussions to the team about how each CNS could feasibly implement HNA activity into their work plan. Conversations about CNS workload also helped the NC understand why despite making improvements in HNA activity after treatment from 29%–42% during 2016, this was not sustainable in 2017.

The NC was cognisant of other barriers the CNSs faced in trying to support patients LWBC; for example, not being able to refer individuals with significant emotional distress to a dedicated psycho‐oncology service proved a disincentive to exploring emotional concerns. It was possibly easier for a NC as a clinician, rather than a project manager, to support discussions on such matters in the meetings – in this case the therapeutic value of the assessment and the other options available to support people in distress. These suggestions were perhaps more readily accepted when delivered by someone who was similarly managing these difficulties in their own clinical practice.

The personal reflection below in Table 8 provides further insights into this process.

**Table 8:** Personal reflection June 17

Personal reflections and summary
Achieving a comprehensive service to those LWBC requires a considerable cultural shift within the Trust – and this takes time. I aimed to influence by offering senior leadership to the cancer nursing team and being a role model to others. I have both great persistence and enthusiasm, but it took longer than expected for people to own these changes. The lack of administrative support and IT resource available initially certainly delayed implementation. Nonetheless, I believe we have done well in implementing most aspects of the Recovery Package in the Trust to some extent. I consider my knowledge and understanding of survivorship has been a positive influence on team development. Further work is needed in order to align practices across teams and instil individual clinician accountability

## 4 DISCUSSION

The evidence presented demonstrates how the NC role contributed to the implementation of the RP to the Trust and the wider activities undertaken to influence the LWBC agenda regionally and nationally. The findings are now evaluated using the Donabedian model using the three components: structure, process and outcomes.

### 4.1 Structure – input measures

At the time of the NC’s role commencement, the Cancer CNS team had a low level of awareness of the work of the National Cancer Survivorship Initiative and contribution they could make to the needs of those living with and beyond cancer. Only a third of the teams had started to offer HNAs by using paper copies of the tool. There was no recording of any of this RP activity by the CNSs as no team had any administration assistance or support worker. The Breast Care team were running an end of treatment nurse‐led clinic, but the consultation did not include HNA or TSs, and there was concern about a high non‐attendance (DNA) rate with risk of patients being lost to follow‐up. Patients seeking further support after completing cancer treatment in the Trust could access a patient support programme twice in one year facilitated by the Penny Brohn charity. The team started offering their own HOPE (Life after cancer) programme in February 2016 and an established HWBE schedule in June 2016 (previous attempts have been made to run HWBEs, but they were still in evolving up until this point).

By the end of the evaluation time period, review of the structural changes to the team highlighted that many of the site‐specific CNSs had made substantial and sustained advances in their nursing practice, integrating HNAs into their usual care and inviting all their patients to HWBEs. The Breast CNS team formalized their end of treatment offer to ensure all patients received a HNA, TS, after‐care information and offer of a HWBE. Teams started tracking their patients using excel spreadsheets to ensure no one was lost to follow‐up. The CNS team recognized the role they could play beyond treatment in signposting people LWBC to services in the locality to improve their health and well‐being such as exercise referral schemes.

Certainly, one of the main influencers for the development of the LWBC service to the Trust was the addition of the support workers (SW) to the cancer nursing team. The SWs were instrumental to the running of the HWBEs and HOPE programme. The NC, the Breast Senior CNS and the Lead nurse all provided the SWs with valuable support and training opportunities; however, it was the NC that led recruitment to these posts and monitored their performance. As their experience developed and confidence increased, their reliance on the NC lessened so that by September 2017, the SWs were working well as a team and could lead the HWBE without seeking weekly guidance from the NC. Their contribution to RP implementation was significant.

### 4.2 Process – the way systems and processes work to deliver the desired outcome

The NC helped drive and support RP activity by providing bespoke educational events, securing additional resources, for example IT applications, agreeing a HWBE budget, developing localized patient information and chairing the quarterly LWBC senior CNS meetings. The NC particularly focused on supporting the “early adopters” in the three largest CNS teams by offering positive feedback and encouragement to further relevant nurse‐led activity. She made a direct contribution to RP activity by undertaking HNAs with her patient group, at the two time points.

The NC organized and facilitated the running of HWBE through this time and whilst numbers started to grow, activity remained under target until support workers were recruited. By the end of the evaluation period, all CNSs were involved in these events, independently led sessions to the agenda and took responsibility for recruiting their patients to them. The NC was dominant to creating this embryonic service change whereby all the team became engaged and enthusiastic about these events. Sharing the positive patient evaluations soon after each event with all the team and praising the staff involved was one important strategy.

Engaging clinical teams to complete treatment summaries (TSs) by providing education, resources and personal support proved more challenging. The breast CNS team fully embraced TS implementation appreciating how it could improve communication between both patient and GPs and personalize after‐care. However, across the other site‐specific cancer teams, TS activity remained low despite the NC briefing all (MDTs). On reflection, the NC may also have achieved greater engagement across the multidisciplinary team had she managed to get the LWBC agenda on to the Trust Cancer Board agenda sooner (since this did not happen until later in 2017), making it a quality improvement measure relevant to all. Nonetheless, it could also be argued that the target set – 70% of patients to receive a TS on completing treatment – is unrealistic since the implementation of TSs has been similarly slow across all Trusts in the Cancer Vanguard with none reaching even half the target number at Q1 2017/8.

The 360–degree feedback highlighted the NC’s passion for promoting service development but due to other role demands, perhaps insufficient time was devoted to project management to meet all targets set. Yet, even in Trusts that employed a full‐time LWBC project manager many of these targets were not met reflecting the complexity of this change process (Doyle, 2019; Greenfield & Proctor, 2018; RMPartners, 2018).

The NC made a considerable investment through both academic publications (*N* = 7), national presentations (*n* = 4) and production of patient information/guidance for national charities (>10) during this time which added to the knowledge base of LWBC for both patients and healthcare providers. Considerable time was spent working at a regional and national level to effect change at a wider, strategic level.

### 4.3 Outcomes – impact on the patient and the result of the improvement

The implementation of the RP had direct benefit on patient care in this Trust in three main ways: (a) by providing clarity of and continuity in the care delivered to those completing cancer treatment; (b) by identifying needs through conducting HNAs with subsequent care planning offering a more person‐centred approach; and (c) by enhancing partnership working between the CNSs and patients as they interacted at the HWBE on equal terms.

Redwood et al. (2005) suggests that a NC who offers transformational leadership can help create a context and culture that facilitates the integration of evidence into practice. What is less well described in the published literature is that such leadership can also produce tangible changes in the workplace culture, in this case achieved by the CNS teams working together on a shared goal and more openly discussing issues – often at the LWBC meetings – promoting wider collaboration and peer support (Manley, Sanders, Cardiff, & Webster, 2011).

There are perhaps less obvious benefits from the NC’s wider influencing of the LWBC strategic agenda and the outcomes from such activity are harder to discern. Peer feedback in the survey identified the tension that NCs can have in striking a balance between local work and regional work as time spent away from the Trust meant the NC was not as available and visible as some would have liked. This view might however reflect a lack of appreciation that a NC role should be multidimensional, diverse and take on broader strategic roles (Duffield et al., 2011; Por, 2008).

It is difficult to state how much the NC role alone may have influenced this change process since health care is delivered in complex systems and any influence the NC had did not happen in isolation from the interactions between the other individual components (Carballo, 2016). For example, the importance of having administrative support in this improvement process was underestimated at the outset – our activity increased once the SWs employed.

In line with Leary, Whittaker, & Hill (2017), the cancer CNSs in this Trust felt overworked and some resisted this “change process” due to fear of the additional work demand. By seeing their concerns from the “inside,” it was perhaps easier for the NC to work alongside them and find solutions. Also, the NC’s direct role in delivering these interventions not only enhanced her credibility but also enabled effective role modelling (Redwoo et al., 2005).

The NC was conscious of the pressures on the Trust to meet the 62‐day target (promising patients a start date for their cancer treatment within 62 days of an urgent referral being received) but was keen that operational demands did not dominate the senior management agenda at the expense of quality improvements, predominantly achieved by educating clinicians, challenging attitudes to LWBC and promoting concepts such as supported self‐management and patient‐centred after‐care. Embedding new mindsets takes time but is necessary precursors to transforming roles and responsibilities (Giles et al., 2014; Kearney, Miller, Paul, Smith, & Rice, 2003). NCs are well‐placed and can act as a powerful force in creating and sustaining change at the patient–provider interface to enhance the quality of the patient care provided (Kitson, 2001; The King’s Fund, 2018). The findings presented in this reported evaluation are reflective of those from Kennedy et al's review (2012) of 36 studies which suggest the largely positive influence NCs can have on a range of clinical and professional outcomes.

Nevertheless, NC role outputs are dependent to a large extent on the skills and experience possessed (Gerrish et al., 2011; Pottle, 2018). This NC was new in role but did have considerable expertise in the specialty, experience in facilitating individual/team learning, research approaches and transformational leadership (Manley, 1997, 2000, 2002). Role achievement is also dependent on organizational support and prevailing Trust culture if nurses are to function at their highest capacity (Fernandez, Shepppard‐Law, & Manning, 2017; McCorkle et al., 2012). Since this Trust's stated priority is patient care, the NC’s clinical workload always took priority over service development, education and research. Protecting time for these other essential aspects of the NC role is a known and commonly reported challenge (Dyson, 2014).

Future research is needed to demonstrate the importance of NCs maintaining all four of the role functions that define this highly specialized role, over and above the provision of expert practice; professional leadership and consultancy, education, training and development practice and service development and research and evaluation.

## 5 LIMITATIONS

This evaluation used four different methods together to look at the various influences of a NC in one team with respect to the LWBC agenda. Ideally, data sources should have included more patient feedback with just 3 patients included in the 360‐degree feedback process. However, individual patients would not have been able to comment on how service delivery might have improved over time. Additional external appraisals might have made the feedback more objective, but there was insufficient funding to do this.

Another difficulty proved to be extrapolating the unique contribution the NC may have made as just one individual to a multidisciplinary team of a complex healthcare system Richardson, Ainsworth, Humphreys, Stenhouse, and Watkins (2008) concur and suggest that this is best addressed through the use of both quantitative and qualitative approaches, as undertaken here. Overall, the four evaluation questions have been answered and the aims of this role evaluation have been met using the methods described.

## 6 CONCLUSIONS

This evaluation of a NC role in one important aspect of practice – living with and beyond cancer – has highlighted the nature of contribution that it can offer not just to patient care but also in broader ways by helping shape delivery of after‐care in one secondary cancer service whilst also advancing professional understanding at both a regional and national level. It has indicated ways the NC was able to directly and indirectly enhance outcomes over two and a half years in post, with evidence of a continued commitment to achieving results over the evaluation period.

In working as a transformational leader to her team, the NC drove a vision of care needed not just to meet targets but ultimately to improve the lives of those LWBC. She inspired others to deliver better care and developed a culture where the needs of patients remained at the heart of clinical care. Consequently, the Recovery Package became embedded into working practice and delivery of its core interventions became “usual care.”

## 7 RELEVANCE TO CLINICAL PRACTICE

There is increasing pressure on senior nurses to identify and measure their individual impact on patient outcomes. The outcomes reported by this evaluation illustrate the influence that the NC can have not just only on the development of services but also on workplace culture. NCs must be clear about their scope, responsibilities and core functions if they are to successfully articulate their value. The use of a framework which highlights connections between structure, process and outcome may help make their role contribution to the healthcare team more discernible.

## CONFLICT OF INTEREST

The authors have no competing interests.
